# Validation of the loop-mediated isothermal amplification method for rapid and sensitive detection of *Ureaplasma* species in respiratory tracts of preterm infants

**DOI:** 10.1371/journal.pone.0247618

**Published:** 2021-03-04

**Authors:** Yuta Mikami, Kazumasa Fuwa, Eriko Arima, Yasuo Suda, Itaru Yanagihara, Satoshi Ibara

**Affiliations:** 1 Department of Neonatal Medicine, Kagoshima City Hospital, Kagoshima, Japan; 2 Department of Creation of Community Medicine, Graduate School of Medical and Dental Sciences, Kagoshima University, Kagoshima, Japan; 3 Department of Pediatrics and Child Health, Nihon University School of Medicine, Itabashi, Tokyo, Japan; 4 Department of Chemistry, Biotechnology and Chemical Engineering, Graduate School of Science and Engineering, Kagoshima University, Kagoshima, Japan; 5 Department of Developmental Medicine, Research Institute, Osaka Women’s and Children’s Hospital, Osaka, Japan; Seoul National University College of Medicine, REPUBLIC OF KOREA

## Abstract

**Introduction:**

A simple and rapid diagnosis of *Ureaplasma* spp. is required for the choice of the appropriate antibiotic. However, an ideal detection method has not been available. This study examines the efficacy of the loop-mediated isothermal amplification (LAMP) assay, which provides rapid and sensitive results, to detect *Ureaplasma* spp. in respiratory tract samples of preterm infants.

**Methods:**

The study included preterm infants born before 32 weeks of gestation admitted Kagoshima City Hospital from June 2018 to March 2020. Nasopharyngeal swabs and/or tracheal aspirates were obtained in the first seven postnatal days. One hundred sixty-seven nasopharyngeal swabs and 101 tracheal aspirates were analyzed by LAMP, culture, and quantitative real-time polymerase chain reaction.

**Results:**

All 167 infants had a median (range) gestational age of 28.7 weeks (22.3–30.9) and birthweight 1030g (322–1828). One hundred sixty-seven nasopharyngeal swabs and 101 tracheal aspirates were obtained. In the results of nasopharyngeal swabs, the sensitivity and specificity of LAMP were 73.9% (17/23) and 97.2% (140/144), whereas those of quantitative real-time polymerase chain reaction were 73.9% (17/23) and 95.8% (138/144), compared to culture. In the results of tracheal aspirates, the sensitivity and specificity of LAMP were 89.5% (17/19) and 92.7% (76/82), whereas those of quantitative real-time polymerase chain reaction were 89.5% (17/19) and 93.9% (77/82), compared to culture.

**Conclusions:**

The LAMP assay showed similar sensitivity and specificity with quantitative real-time polymerase chain reaction in the respiratory tracts of preterm infants including extremely preterm infants during the immediate postnatal period. Therefore, the LAMP is a practical alternative for the early detection so that appropriate antibiotics can be administered for preventing BPD.

## Introduction

*Ureaplasma* species, which belong to the *Mycoplasmataceae* family of bacteria, are self-replicating minimal cells that lack a cell wall. Two biovars, *Ureaplasma parvum* (*U*. *parvum*) and *Ureaplasma urealyticum* (*U*. *urealyticum*), are most often found in humans. *Ureaplasma* spp. are frequently isolated from the amniotic fluid and placentas of preterm infants [1, 2] and have been associated with adverse prenatal outcomes such as miscarriages, preterm labor, preterm premature rupture of membranes, and chorioamnionitis [3].

In preterm infants, the rate of respiratory colonization with *Ureaplasma* spp. has had a positive correlation to prematurity [4]. Three meta-analyses reported a significant association between *Ureaplasma* respiratory colonization and bronchopulmonary dysplasia (BPD) [5–7]. Animal studies also support the hypothesis that *Ureaplasma* colonization can lead to lung injury and BPD [8, 9].

Although ampicillin and gentamicin are usually prescribed to preterm infants prophylactically, *Ureaplasma*, which does not have a cell wall, is resistant to these antibiotics and responds best to macrolides for prevention of BPD [10, 11]; one meta-analysis assessing the use of macrolides reported that azithromycin is effective against BPD alone and BPD and/or death [12].

A simple and rapid diagnosis of *Ureaplasma* spp. is not currently available, which hampers clinicians in correctly choosing the most effective antibiotic. A bacterial culture, the gold–standard method, takes two to three days and requires special media and a culture chamber [13]. Polymerase chain reaction (PCR) can be an alternative, but three hours are required to purify and amplify the DNA; PCR can be performed only in research laboratories due to the necessity for a thermal cycler.

The loop-mediated isothermal amplification (LAMP) assay is an alternative nucleic acid detection method that uses a unique priming mechanism [14]. The DNA amplification reaction occurs in an isothermal condition without thermal cycling, and the unaided eye can recognize LAMP-amplified products by observing the turbidity of the reaction tubes; these advantages could enable bedside use of the LAMP assay to detect *Ureaplasma* spp.

Fuwa et al. developed a novel detection method for *Ureaplasma* spp. using the LAMP method and demonstrated that it outperformed the culture test and conventional PCR method in vaginal swab samples [15]. However, it has not been demonstrated if this method would be effective for neonates. This study compares the LAMP assay, quantitative real-time PCR (qPCR), and cultures to detect *Ureaplasma* spp. in respiratory tract samples of preterm infants.

## Materials and methods

### Clinical specimens

The Institutional Review Board of Kagoshima City Hospital approved the study protocol (Approval Number: 2017–57). Written parental consent was obtained for all participants. Eligible subjects were preterm infants born before 32 weeks gestational age and admitted to the neonatal intensive care unit of Kagoshima City Hospital (Kagoshima, Kagoshima, Japan) from June 2018 to March 2020. The specimens were obtained in the first seven postnatal days and transported to the laboratory within three hours at room temperature. Nasopharyngeal swabs and tracheal aspirates were simultaneously obtained from infants who were mechanically ventilated; only nasopharyngeal swabs were obtained from non-intubated infants. Two FLOQ swabs (Becton Dickinson, Franklin Lakes, NJ, US) were obtained from each infant: one for the bacterial culture, and the other suspended in 200 μL saline for qPCR and LAMP detection.

DNA was extracted for the LAMP assay by an SR DNA extraction kit (Eiken Medical Co., Ltd., Tokyo, Japan) and for qPCR assay by a QIAamp DNA mini-kit (QIAGEN, Valencia, CA, US), according to the instructions of manufacture. The tracheal aspirates were obtained by suctioning with TRACHCARE (Halyard Health, Alpharetta, TX, US). If the yield was insufficient, 0.5–1 ml of saline was installed intratracheally before suctioning again. The tracheal aspirates were centrifuged for 15 minutes at 20,000 g. After removal of the supernatant, the remaining 200 μL was used for culture and DNA extraction. All primers were synthesized by Hokkaido System Science (Sapporo, Hokkaido, Japan).

### Preparation for positive controls

The cloned fragments of the *ureB* gene of *U*. *parvum* (SV3F4) [16] and *U*. *urealyticum* (UU3) [17] were used as a positive control for each *Ureaplasma* species. The cloning procedure for *ureB* genes was performed in accordance with a previous report [15].

### LAMP reaction

LAMP primers targeting the *ureB* gene for the detection of *U*. *parvum* or *U*. *urealyticum* were taken from the published study by Fuwa et al. (S1 Table) [15]. LAMP reactions were performed in a 25-μl volume consisting of 5 μl template, 8 U of *Bst* DNA polymerase, 25 mM deoxynucleoside triphosphates, 4 M betaine, 1.5 M Tris-HCl (pH 8.8), 2.5 M KCL, 1 M (NH_4_)SO_4_, 1 M MgSO_4_, and 20% Tween 20. Primers were used at these concentrations: 1.6 μM each of FIP and BIP, 0.2 μM each of F3 and B3, 0.4 μM each) of LF and LB. The mixture was incubated at 63°C for 60 min and then heated at 80°C for 2 min to terminate the reaction. The detection was performed by real-time measurement of turbidity using a Loopamp EXIA (Eiken Medical Co., Ltd., Tokyo, Japan) and visual observation. Every assay included positive *U*. *parvum* or *U*. *urealyticum* DNA and negative controls (nuclease-free water). All samples were tested in duplicate. A positive result in either *U*. *parvum* or *U*. *urealyticum* was defined as a positive LAMP result.

### Quantitative real-time PCR reaction

The qPCR primers targeting the *ureB* gene to detect *U*. *parvum* or *U*. *urealyticum* were taken from the study by Mallard et al. (S1 Table) [18]. The qPCR SYBR Green assay was performed in a 25-μL volume containing 5 μL of template, using a Thermal Cycler Dice Real-Time System (Takara Bio Inc., Shiga, Japan) with a commercial reagent (TB Green Premix Ex Taq II [Tli RNaseH Plus], Takara Bio, Inc., Kusatsu, Shiga, Japan) according to the manufacturer’s instructions. The concentration of each primer was 0.4 μM in the reaction mixture. The qPCR protocol was 45°C for 5 min, 95°C for 10 s, and 45 cycles of 95°C for 30 s, 60°C for 30 s, and 72°C for 45 s.

To determine the specificity of the qPCR reactions, a melt-curve analysis was performed after amplification by slow heating from 60°C to 90°C, with fluorescence acquisition at 0.5°C intervals. The assay result was considered positive when the number of the threshold cycle (Ct) was 38 or less, and the melting temperature (Tm) variation between the sample and the positive control was within 1°C. Every assay included positive *U*. *parvum* or *U*. *urealyticum* DNA and negative (nuclease-free water) controls. All samples were tested in duplicate. A positive result in either the *U*. *parvum* or *U*. *urealyticum* was defined as a positive qPCR result.

### Culture

The specimens were cultured in a Urea-arginine LYO2 broth (Biomerieux, Lyon, France) in 5% CO_2_ at 35°C for 48 hours. Nasopharyngeal swabs were soaked in the broth, and 100 μL of tracheal aspirates were applied to the broth. After the incubation period, the color of the medium changed from yellow to red because of urea hydrolysis, indicating *Ureaplasma* spp. positivity. We identified *U*. *parvum* or *U*. *urealyticum* by LAMP assay, which was performed as described above using extracted DNA from 25 μL of the positive broth.

### Statistical analysis

The clinical sensitivity, specificity, positive predictive values (PPV), and negative predictive values (NPV) of the LAMP and qPCR assays were compared to those of the conventional culture method, which provided the gold standard. The 95% confidential interval (CI) was calculated using a standard interval estimation and Jeffreys prior interval for a small n, as previously described [19]. Data were analyzed using Excel 2013 (Microsoft, Redmond, WA, USA).

## Results

There were 167 preterm infants eligible for the study, and, of those, 101 were mechanically ventilated. The characteristics of both groups are shown in Table 1. All 167 eligible infants had a median (range) gestational age (GA) of 28.7 weeks (22.3–30.9) and birthweight 1030g (322–1828). The 101 intubated infants were GA of 28.9 weeks (22.3–31.7) and birthweight 804g (322–1808). The median day (range) of taking nasopharyngeal swabs was day1 (day0-7). Tracheal aspirates were taken on day1 (day0-7). We collected 167 nasopharyngeal swabs, one from each infant, and 101 tracheal aspirates, one from each of the intubated infants.

**Table 1:** Characteristics of the subjects.

	All eligible infants (n = 167)	Mechanically ventilated infants (n = 101)
Gestational age (weeks)	28.7 (22.3–30.9)	28.9 (22.3–31.7)
Birth weight (g)	1030 (322–1828)	804 (322–1808)
Sex (Male: Female)	89:78	46:55
Apgar score (1min)	4 (1–8)	3 (1–8)
Apgar score (5min)	7 (2–10)	6 (2–9)
day of sample collection	1 (0–7)	1 (0–7)

Data are given as median (range).

The culture positivity in nasopharyngeal swabs and/or tracheal aspirates was 27/167 infants (16.2%). In 27 culture-positive infants, both nasopharyngeal swabs and tracheal aspirates were obtained in 22 intubated infants, and only nasophalyngeal swabs were taken in five non-intubated infants. Among the 22 infants, four infants were culture-positive only in tracheal aspirates and three infants were culture-positive only in nasopharyngeal swabs. In 15 infants who were culture-positive in both samples, *U*. *parvum* was detected in 11 infants, and *U*. *urealyticum* was detected in the remaining four infants. The LAMP assays completely matched in both samples.

To compare the detection of *U*. *parvum* and *U*. *urealyticum*, LAMP assays were performed on the 27 culture-positive infants. *U*. *parvum* was detected in 22 infants, and *U*. *urealyticum* was detected in the remaining five infants. No simultaneous detection of *U*. *parvum* and *U*. *urealyticum* was observed.

### Nasopharyngeal swabs

**Fig 1:**
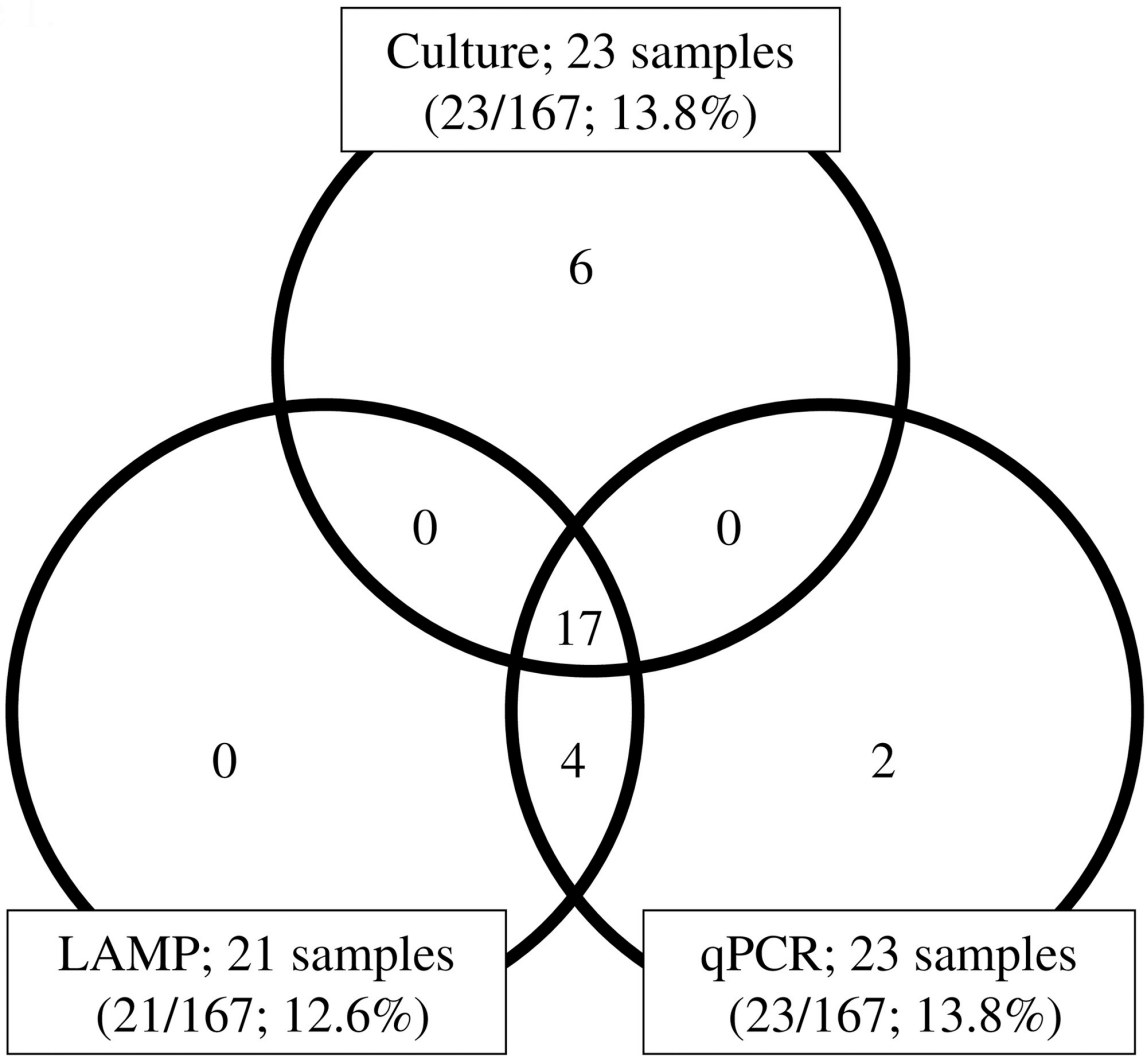
Detection of *Ureaplasma* species in nasopharyngeal swabs analyzed by culture, LAMP, and qPCR. In each field of the venn diagram, the number of samples positive for *Ureaplasma* species is given. LAMP; Loop-mediated isothermal amplification. qPCR; Quantitative real-time PCR.

Twenty-three of the 167 nasopharyngeal samples (13.8%) were culture-positive; 21/167 samples (12.6%) were LAMP-positive; and 23/167 samples (13.8%) were qPCR-positive. Of the 23 culture-positive samples, 17 showed a complete match with LAMP and qPCR results, and six were LAMP-negative/qPCR-negative. Four culture-negative samples were positive by both LAMP and qPCR; two qPCR positive samples were negative by both culture and LAMP (Fig 1).

Table 2 shows the overall performances of the LAMP and qPCR tests to detect *Ureaplasma* spp. from nasopharyngeal samples. The results of LAMP compared to culture were sensitivity: 73.9% (17/23; 95% CI, 53.9–88.3%); specificity: 97.2% (140/144; 95% CI, 93.5–98.3%); PPV: 81.0% (17/21; 95% CI, 60.8–93.2%); and NPV: 95.9% (140/146; 95% CI, 91.7–98.3%). In contrast, the outcomes of qPCR were sensitivity: 73.9% (17/23; 95% CI, 53.9–88.3%); specificity: 95.8% (138/144; 95% CI, 91.6–98.2%); PPV: 73.9% (17/23; 95% CI, 53.9–88.3%); and NPV: 95.8% (138/144; 95% CI, 91.6–98.2%).

**Table 2:** Comparison of LAMP, quantitative real-time PCR, and culture results from 167 nasopharyngeal swab specimens.

Detection of *U*. *parvum* or *U*. *urealyticum*^a^		Culture^b^			Sensitivity (95% CI)	Specificity (95% CI)	PPV^c^ (95% CI)	NPV^d^ (95% CI)
		Pos	Neg	Total				
LAMP^e^	Pos	17 (4)^f^	4 (1)	21 (5)	73.9% (53.9–88.3)	97.2% (93.5–98.3)	81.0% (60.8–93.2)	95.9% (91.7–98.3)
	Neg	6	140	146	17/23	140/144	17/21	140/146
qPCR^g^	Pos	17 (4)	6 (1)	23 (5)	73.9% (53.9–88.3)	95.8% (91.6–98.2)	73.9% (53.9–88.3)	95.8% (91.6–98.2)
	Neg	6	138	144	17/23	138/144	17/23	138/144
	Total	23	144	167				

^a^ *U*. *parvum*: *Ureaplasma parvum*, *U*. *urealyticum*: *Ureaplasma urealyticum*.

^b^ Cultures were performed with Urea-arginin LYO2 broth. Pos indicates that a *Ureaplasma* strain was isolated, and Neg indicates that no *Ureaplasma* strain was isolated.

^c^ PPV: positive predictive value.

^d^ NPV: negative predictive value.

^e^ LAMP reactions were performed using a Loopamp real-time turbidimeter (LA-500; Eiken Co., Ltd.). Pos indicates that amplification occurred in either the *U*. *parvum* or *U*. *urealyticum* LAMP assay, and Neg indicates that amplification did not occur in neither the *U*. *parvum* nor the *U*. *urealyticum* LAMP assays.

^f^ Number of *U*. *urealyticum* culture-positive samples confirmed by LAMP assay. In the 23 culture-positive samples, *U*. *parvum* was detected in 19 samples, and *U*. *urealyticum* was detected in the remaining 4 samples.

^g^ Quantitative real-time PCR (qPCR) was confirmed using a Thermal Cycler Dice Real Time System (Takara Bio Inc.). Pos indicates that amplification occurred with the *U*. *parvum* or *U*. *urealyticum* qPCR assay, and Neg indicates that amplification did not occur in neither the *U*. *parvum* nor the *U*. *urealyticum* qPCR assay.

### Tracheal aspirate

**Fig 2:**
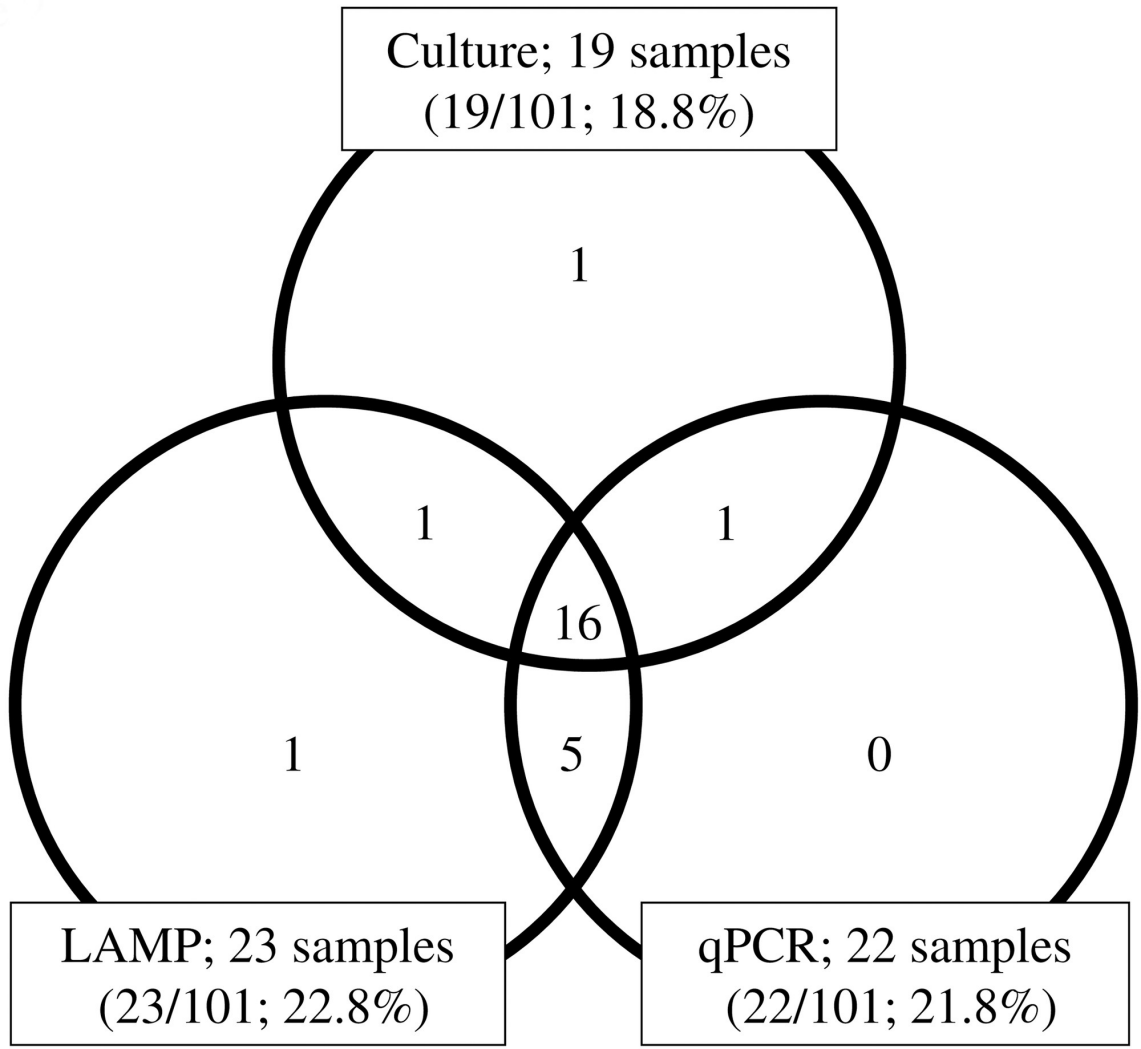
Detection of *Ureaplasma* species in tracheal aspirates analyzed by culture, LAMP, and qPCR. In each field of the venn diagram, the number of samples positive for *Ureaplasma* species is given. LAMP; Loop-mediated isothermal amplification. qPCR; Quantitative real-time PCR.

In 101 tracheal aspirates, 19/101 samples (18.8%) were culture-positive, 23/101 samples (22.8%) were LAMP-positive, and 22/101 samples (21.8%) were qPCR positive. Among 19 culture-positive samples, 16 samples showed a complete match with LAMP and qPCR results; one sample was LAMP-negative/qPCR-negative; one sample was LAMP-positive/qPCR-negative; and one sample was LAMP-negative/qPCR-positive. In five culture-negative samples, all were positive by both LAMP and qPCR. In addition, one LAMP positive sample was negative by both culture and qPCR (Fig 2).

The overall performances of the LAMP, qPCR, and culture tests used for the detection of *Ureaplasma* spp. in tracheal aspirates are shown in Table 3. Comparing LAMP to culture, the results were sensitivity: 89.5% (17/19; 95% CI, 70.3–97.8%); specificity: 92.7% (76/82; 95% CI, 85.5–96.9%); PPV: 73.9% (17/23; 95% CI, 53.9–88.3%); and NPV: 97.4% (76/78; 95% CI, 92.0–99.5%). In contrast, the outcomes of qPCR were sensitivity: 89.5% (17/19; 95% CI, 70.3–97.8%); specificity: 93.9% (77/82; 95% CI, 87.2–97.6%); PPV: 77.3% (17/22; 95% CI, 57.1–90.8%); and NPV: 97.5% (77/79; 95% CI, 92.1–99.5%).

**Table 3:** Comparison of LAMP, quantitative real-time PCR, and culture results from 101 tracheal aspirate specimens.

Detection of *U*. *parvum* or *U*. *urealyticum*^a^		Culture^b^			Sensitivity (95% CI)	Specificity (95% CI)	PPV^c^ (95% CI)	NPV^d^ (95% CI)
		Pos	Neg	Total				
LAMP^e^	Pos	17 (5)^f^	6 (1)	23 (6)	89.5% (70.3–97.8)	92.7% (85.5–96.9)	73.9% (53.9–88.3)	97.4% (92.0–99.5)
	Neg	2	76	78	17/19	76/82	17/23	76/78
qPCR^g^	Pos	17 (5)	5	22 (5)	89.5% (70.3–97.8)	93.9% (87.2–97.6)	77.3% (57.1–90.8)	97.5% (92.1–99.5)
	Neg	2	77	79	17/19	77/82	17/22	77/79
	Total	19	82	101				

^a^ *U*. *parvum*: *Ureaplasma parvum*, *U*. *urealyticum*: *Ureaplasma urealyticum*.

^b^ Cultures were performed in Urea-arginine LYO2 broth. Pos indicates that a *Ureaplasma* strain was isolated, and Neg indicates that no *Ureaplasma* strain was isolated.

^c^ PPV, positive predictive value.

^d^ NPV, negative predictive value.

^e^ LAMP reaction was performed using a Loopamp real-time turbidimeter (LA-500; Eiken Co., Ltd). Pos indicates that amplification occurred in the *U*. *parvum* or *U*. *urealyticum* LAMP assay, and Neg indicates that amplification did not occur in neither the *U*. *parvum* nor the *U*. *urealyticum* LAMP assays.

^f^ Number of *U*. *urealyticum* culture-positive samples confirmed by LAMP assay. In the 19 culture-positive samples, *U*. *parvum* was detected in 14 samples, and *U*. *urealyticum* was detected in the remaining 5 samples.

^g^ Quantitative real-time PCR (qPCR) was confirmed using a Thermal Cycler Dice Real Time System (Takara Bio, Inc.). Pos indicates that amplification occurred with the *U*. *parvum* or *U*. *urealyticum* qPCR assay, and Neg indicates that amplification did not occur in neither the *U*. *parvum* nor the *U*. *urealyticum* qPCR assays.

## Discussion/Conclusion

*Ureaplasma* spp. respiratory colonization has been shown to cause pulmonary inflammation and altered lung development [20] in neonates and is a significant risk factor for BPD [5–7]. Antibiotics that are prophylactically given to preterm infants (ampicillin and gentamicin) are ineffective against *Ureaplasma*, but studies have reported that using macrolides antibiotic therapy reduced the incidence of BPD [10–12]. Hence, it is important to develop a simple and rapid method to detect *Ureaplasma* spp. in preterm infants. This study clarified that the accuracy of the LAMP method to detect for *Ureaplasma* spp. was similar with that of qPCR. The LAMP method was most sensitive in the tracheal aspirates from intubated preterm infants, who are at high risk of developing BPD.

We studied preterm infants of less than 32 weeks of gestational age including extremely preterm infants. All samples were taken during immediate postnatal period. The culture positivity in nasopharyngeal swabs and/or tracheal aspirates was 27/167 infants (16.2%). The detection rate of *Ureaplasma* spp. in our study was similar to the results found in previous studies. In a study by Ballard et al., the culture-based detection rate in the tracheal aspirates of intubated infants weighing less than 1,000 g was 18.6% (8/43) [21]. In a study by Jonsson et al., the detection rate by culture among intubated infants of less than 30 weeks gestational age was 15.5% (24/155) in bronchial aspirates and 10.3% (16/155) in nasopharyngeal swabs [22]. In a study by Lyon et al., *Ureaplasma* spp. were detected in tracheal aspirates by both culture test method and conventional PCR in 15.0% (9/60) of infants at 30 or fewer weeks of gestational age [23].

Bacterial cultures are still the gold standard for the detection of *Ureaplasma* spp., and have the advantage of performing drug sensitivity tests and detecting other pathogens [13]. However, it takes at least 2–3 days and more to analyze drug sensitivities. Antibiotic therapy for BPD prevention should not be delayed after the results are available, as a pro-inflammatory response in the developing lung occurs even in utero [20].

Detecting *Ureaplasma* spp. with qPCR has been reported to have higher sensitivity and specificity compared to culture. In a study using amniotic fluid samples, the sensitivity and specificity of two qPCRs targeting 16S rRNA were 81.8–92.3% and 89.4–97.4%, respectively [24]. In another study of vaginal swab samples, the sensitivity was 95.1%, and specificity was 75.5% [25]. The low specificity and numerous false positives suggest that a pathogen undetected by culture can be detected by qPCR. However, the qPCR requires a thermal cycler and is not suitable for bedside use.

As an alternative to qPCR, LAMP, a nucleic acid amplification assay, is faster, less complicated, and highly specific [14]. LAMP has been clinically applied to detect various pathogens [26–28], and a standard-displacing DNA polymerase enables isothermal amplification without the thermal cycler. The amplification efficiency and specificity are high because four to six primers are designed for the six gene regions. The amplification process, therefore, is usually completed within one hour, and the results can be visually confirmed. The high amplification efficiency makes it possible to use a simple nucleic acid extraction kit and tolerate the polymerase inhibitors in samples.

Few studies have examined the efficacy of the LAMP assay to detect *Ureaplasma* spp. We recently reported that the LAMP method for *Ureaplasma* spp. was effective to test vaginal swabs [15]. To the best of our knowledge, the present work is the first report on the LAMP method in neonatal samples. We believe our system is superior compared to culture and a qPCR because of its convenience, simplicity, and accuracy.

It is more difficult to collect samples from preterm infants than from adults. A nasopharyngeal swab is hard to introduce into the narrow nasal cavity of preterm infants and can cause bleeding. Sputum can easily be collected from adults, but tracheal intubation and suction are necessary to obtain specimens from the lower respiratory tracts of preterm infants. This procedure, even administered for a short time, can induce bradycardia, hypoxemia, and tachycardia. In neonatal intensive care unit, less-invasive and less-time consuming laboratory tests are preferable because those tests enable neonatologists to address the time margin to select appropriate antibiotics which may prevent BPD.

We found the LAMP assay to be more sensitive in testing bronchial aspirates than in testing nasopharyngeal swabs. The amount of DNA included in some nasopharyngeal swabs may be below the detection limit because of the narrow of nasal cavities and blood contaminations may affect the DNA extraction and amplification. Since the risk of BPD is greater in intubated preterm infants than in non-intubated pretem infants [22], it is reasonable to perform the LAMP method for tracheal aspirates in intubated preterm infants. Nasopharyngeal swabs can be an alternative in case of severe complications during suction.

This study is not without its limitations. First, the PPV of the LAMP method is low. The prevalence of *Ureaplasma* spp. colonization in neonatal respiratory tracts is 10.3–18.7% [21–23]. At the prevalence of *Ureaplasma* spp. colonization, the PPV decreases, and false positives increase even if both the sensitivity and specificity are high. Another limitation is that the LAMP method can detect dead bacteria that are not detectable by culture tests; therefore, it is better to perform culture simultaneously with the LAMP method and confirm whether both results match. Second, this method requires two LAMP tests for both *U*. *parvum* and *U*. *urealyticum*. Our group is currently designing a primer that can detect both *U*. *parvu*m and *U*. *urealyticum* in a single assay. Third, if gene mutations occur in the LAMP target gene regions, the sensitivity may decrease because LAMP method requires 2 to 3 sets of primers compared to a single set of primers used in usual PCR method. The final limitation is the small sample size used from a single institution; studying the LAMP assay in preterm infants would benefit from tests in a multicenter trial with large sample sizes.

In conclusion, we established a rapid and sensitive LAMP method, which was applicable for detecting *Ureaplasma* spp. in preterm infants during the immediate postanatal period.

## Supporting information

S1 Table LAMP primer sequences and quantitative real-time PCR primer sequences in this study. (DOCX)
