# Supplementary material for: Validation of the loop-mediated isothermal amplification method for rapid and sensitive detection of Ureaplasma species in respiratory tracts of preterm infants
Source: PLoS One. 2021 Mar 4;16(3):e0247618. doi: 10.1371/journal.pone.0247618 (PMC7932158; doi:10.1371/journal.pone.0247618)
Supplement: S1 Table — (DOCX) [file pone.0247618.s001.docx]

S1 Table. LAMP primer sequences and quantitative real-time PCR primer sequences in this study

| ***Ureaplasma parvum* LAMP primer (Sequence 5'-3')** | | **Length**  **(base pairs)** |
| --- | --- | --- |
| Up_F3 | TCAAGTCAATTTAGTCCAGGTA | 22 |
| Up_B3 | GGAATATCGAAACGTCGTCC | 20 |
| Up_FIP | GACGGTCCCCAGTATTTTTAATACTGCAATTAATTTCGCTAGTGGTG | 47 |
| Up_BIP | CAAGTTGGATCACATTTTCACTTGTGCGTTCTTTATCTTCATTTCCTT | 48 |
| Up_LF | AATTACTTTTGCCTCTCTACC | 21 |
| Up_LB | TGAAGTGAATAGTGCATTAG | 20 |
| ***Ureaplasma urealyticum* LAMP primer (Sequence 5'-3')** | |  |
| Uu_F3 | GGTAAATTAGTACCAGGAGCA | 21 |
| Uu_B3 | AACGACGTCCATAAGCAA | 18 |
| Uu_FIP | AGGACGGTCACCAGTATTTTTAAT-ATTAACTTCGCTGAAGGCG | 43 |
| Uu_BIP | CCAAGTTGGATCACATTTCCACTT-CGTTCTTTGTCTTCGTTTCC | 44 |
| Uu_LF | GCTTCTCTACCTTCGTTCAT | 20 |
| Uu_LB | AGTGCATTAGTATTCTTTGATGA | 23 |

| ***Ureaplasma parvum* quantitative real-time PCR primer (Sequence 5'-3')** | | **Length**  **(base pairs)** |
| --- | --- | --- |
| UPF | GAT CAC ATT TTC ACT TGT TTG AAG TG | 26 |
| UPR | AAC GTC GTC CAT AAG CAA CTT TG | 23 |
| ***Ureaplasma urealyticum* quantitative real-time PCR primer (Sequence 5'-3')** | |  |
| UUF | GAT CAC ATT TCC ACT TAT TTG AAA CA | 26 |
| UUR | AAA CGA CGT CCA TAA GCA ACT TTA | 24 |
